# Phytochemical profiles of edible flowers of medicinal plants of *Dendrobium officinale* and *Dendrobium devonianum*

**DOI:** 10.1002/fsn3.2602

**Published:** 2021-10-04

**Authors:** Ming Zhao, Jiakun Fan, Qianting Liu, Hui Luo, Qingyan Tang, Chongping Li, Jurun Zhao, Xinfeng Zhang

**Affiliations:** ^1^ National‐Local Joint Engineering Research Center on Germplasm Innovation & Utilization of Chinese Medicinal Materials in Southwest China Yunnan Agricultural University Kunming China; ^2^ The Key Laboratory of Medicinal Plant Biology of Yunnan Province Yunnan Agricultural University Kunming China; ^3^ College of Tea Science Yunnan Agricultural University Kunming China; ^4^ College of Food Science and Technology Yunnan Agricultural University Kunming China; ^5^ Longling Institute of Dendrobium Baoshan China; ^6^ China State Key Laboratory of Subtropical Silviculture Zhejiang A&F University Hangzhou China

**Keywords:** *Dendrobium devonianum*, *Dendrobium officinale*, edible flowers, fatty acids, metabonomics, phytochemicals

## Abstract

The discovery of new edible flowers that are nontoxic, innocuous flowers having human health benefits, surveys of their phytochemicals and utilization are of great scientific and commercial interest. *Dendrobium officinale* and *Dendrobium devonianum* are precious Traditional Chinese Medicine. During the massive commercial cultivation, a lot of flowers were produced and certified as edible flowers, and the phytochemical profiles and bioactivities warrant evaluate. The present study aimed to investigate the phytochemicals and antioxidative activities in flowers of *D. officinale* (DOF) and *D. devonianum* (DDF). In total, 474 metabolites were identified using a widely targeted metabonomics method, 16 amino acids and 6 flavonoids were measured using high‐performance liquid chromatography (HPLC), and 8 fatty acids were detected using gas chromatography–mass spectrometry (GC‐MS). Both flowers contained various amino acids, including 7 essential amino acids, diverse flavonoids, especially quercetin, kaempferol and their derivatives, and high levels of methyl linoleate and methyl linolenate. The relative levels of quercetin, kaempferol and their glycosides were higher in DDF than in DOF, whereas the relative levels of several flavonoids C‐glycosides were high in DOF. Ethanol extracts of both DOF and DDF showed antioxidative capacities including the scavenging of 1,1‐diphenyl‐2‐picrylhydrazyl and hydroxyl radicals. Both edible flowers contained flavonoids, amino acids, and fatty acids and have antioxidative activities, which should be explored for use in functional foods and pharmaceuticals.

## Graphical abstract

*Dendrobium officinale* and *Dendrobium devonianum* are precious Traditional Chinese Medicines. During the massive commercial cultivation, a lot of flowers were produced and certified as edible flowers, and the phytochemical profiles and bioactivities warrant evaluate. The present study aimed to investigate the phytochemicals and antioxidative activities in flowers of *D. officinale* (DOF) and *D. devonianum* (DDF).Both edible flowers contained flavonoids, amino acids and fatty acids, and have antioxidative activities, which should be explored for use in functional foods and pharmaceuticals.

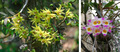

## 1 INTRODUCTION

Edible flowers are defined as nontoxic, innocuous flowers having human health benefits when consumed (Lu et al., 2016). As vegetable, fruit and aromatic flowers (Zhao et al., 2019), they have a long consumption history in many places, such as Asia, ancient Greece and Rome, Medieval France, Europe, Victorian England and the Middle East (Pinakin et al., 2020). Recently, there has been an increasing demand for edible flowers worldwide because of their unique flavors, aromas, and colors, as well as their nutritional values, being rich in carbohydrates and proteins, but low in lipids. They also contain interesting amounts of dietary minerals, such as calcium, iron, potassium, magnesium, phosphorous, and zinc (Pinakin et al., 2020). Furthermore, they are sources of a variety of biologically active low molecular phytochemicals, including phenolic compounds, carotenoids, and tocols, and they possess numerous health benefits, including antianxiety, anticancer, antidiabetic, antiinflammatory, antioxidant, diuretic, anthelmintic, immunomodulatory, and antimicrobial actions (Skrajda‐Brdak et al., 2020).

Edible flowers can be obtained from 97 families, 100 genera, and 180 species worldwide, and the number of edible flowers varies among locations (Lu et al., 2016). Several edible flowers, such as rose (*Rosa* spp.), calendula (*Calendula officinalis*), saffron (*Crocus*), violet (*Viola odorata*), dandelion (*Taravacum officinale*), breaded elder (*Sambucus nigra*), chrysanthemum (*Chrysanthemum coronarium*), daylily (*Hemerocallis fulva*), lilac (*Syringa vulgaris*), mint (*Mentha* spp.), nasturtium (*Tropaeolum majus*), pansy (*Viola wittrockiana*), and tulip (*Tulipa* spp.) have been used or are becoming increasingly popular (P. F. Guiné et al., 2020; Skrajda‐Brdak et al., 2020). The discovery of new edible flowers, surveys of their phytochemicals and the full utilization of known edible flowers are of great scientific and commercial interest.

With more than 1500 species, *Dendrobium* is the second largest genus in Orchidaceae (Yan et al., 2015; Zheng et al., 2018), and its members are widely distributed throughout Asia, Europe, and Australia (Zhao et al., 2019). Among them, 74 species and two varieties are native to China, and the fresh or dried stems of many *Dendrobium* species are used in Traditional Chinese Medicine (Cheng et al., 2019), including those of *Dendrobium officinale* Kimura et Migo, *Dendrobium nobile* Lindl., *Dendrobium huoshanense* C. Z. Tang et S. J. Cheng, *Dendrobium chrysotoxum* Lindl., *Dendrobium fimbriatum* Hook., and related species, which are all officially listed in the Pharmacopoeia of the People's Republic of China. Additionally, *Dendrobium* is traditionally used as an ingredient in food and tea, and the phytochemical profiles of *Dendrobium* represent new resources for specialty cosmetics (Kanlayavattanakul et al., 2018).

At present, the majority of Dendrobium species are propagated by tissue culture and cultivated in massive commercial artificial shelters (Da et al., 2015). For example, in China, the planting acreage of *D. officinale* was >7000 ha in 2016, with the greatest areas occurring in Yunnan, Zhejiang and Guangdong provinces (Guo et al., 2020). Because of the increased planting acreage of *Dendrobium*, large numbers of flowers are produced annually. Thus, the processing and utilization of flowers of *Dendrobium*, and their active components, are also of concern. The flowers of *Dendrobium* contain many nutrients and active ingredients, including naringin, anthocyanins and other flavonoids, polysaccharides, amino acids, and dendrobine, and they have shown antioxidant, liver protection, hypoglycemic and antihypertensive effects (Lai et al., 2017). In a previous work, 19 compounds including 2 phenylpropanoids, 11 C‐glycosylflavones and 6 O‐glycosylflavones, were identified and used as indicators in quantitative evaluations of the quality and authenticity of the flowers of *D. officinale* (DOF) (Zhang et al., 2019). Polysaccharides with antioxidative activities found in flowers of *Dendrobium devonianum* (DDF) have been extracted and characterized (Wang et al., 2018). The crude and partitioned extracts, as well as in the fermented medium, of *Dendrobium sabin* flowers show very good antioxidative properties (Abu et al., 2017).

The flowers of *Dendrobium* have been traditionally used for cooking and tea (Yan et al., 2021). Previously, a beverage using DOF and tea were developed in our lab (Liu et al., 2020). Recently, local food safety standards for DOF in Zhejiang (DB33/3011‐2020), Fujian (DBS35/001‐2020), Guangxi (DBS45/062‐2019) and Guizhou (DBS52/045‐2020) provinces were implemented. In addition, local food safety standards for DOF and DDF in Yunnan Province are being developed. Thus, DOF and DDF are recognized as edible flowers. However, little is known about the phytochemical components in DOF, and the phytochemicals in DDF have not been surveyed.

In this work, to provide a basis for the utilization of DOF and DDF, the total polyphenol contents, as well as the levels of 6 flavonoids, quercetin, taxifolin, rutin, luteolin, kaempferol, and myricetin, were determined. In addition, the 36 fatty acids and 16 amino acids, as well as their antioxidative activities, in DOF and DDF were investigated. A targeted metabolomics analysis was further performed to identify the phytochemicals and compare their levels in these two edible flowers.

## 2 MATERIALS AND METHODS

### 2.1 Chemicals and reagents

Both DOF and DDF were collected at the Longling Institute of Dendrobium (98.696°E, 24.593°N) in May 2019. The reference standards with 98% purity including taxifolin, rutin, myricetin, quercetin, luteolin, and kaempferol were obtained from ChengduMust Biotechnology Co, Ltd Company (Chengdu, China). Acetonitrile (ACN) and methanol (MeOH) for high‐performance liquid chromatography (HPLC) analysis and for the ultra‐performance liquid chromatography–tandem mass spectrometry (UPLC‐MS/MS) analysis were purchased from Merck (Darmstadt, Germany). Deionized water was prepared using a Smart‐Q30 purification system (Hitech Instruments Co., Ltd., Shanghai, China). Other reagents were of analytical grade.

### 2.2 Analysis of chemical compounds in flowers by HPLC or spectrophotometric methods

The total polyphenols and free amino acid contents in flowers were measured using a spectrophotometric ferrous tartrate method (Turkmen et al., 2006) and ninhydrin colorimetric method, respectively. The taxifolin, rutin, myricetin, quercetin, luteolin, and kaempferol contents in the flowers were determined using an Agilent 1200 series HPLC system (Agilent Technologies, Santa Clara, CA, USA). Briefly, flowers were finely powdered. In total, 1 g of powder was extracted with 44.00 ml of methanol: hydrochloric acid (40:4, v/v) in a flask equipped with a reflux condenser. The extraction was performed in a water bath (at 85℃) for 90 min. The extractions were diluted to 50 ml and filtered through a 0.2‐μm nylon filter and then analyzed directly by HPLC as described in our previous work (Nian et al., 2019).

The amounts of amino acids, including alanine, arginine, aspartic acid, cystine, glutamic acid, glycine, histidine, isoleucine, leucine, methionine, phenylalanine, proline, serine, threonine, tyrosine, and valine, in flowers of Dendrobium were measured using a high‐performance liquid chromatography with fluorescence detection (HPLC–FLD) method with online o‐phthalaldehyde (OPA) derivatization in accordance with our previous work, with modifications (Zhao et al., 2011). Briefly, 1 g of flower powder was extracted with 100 ml of distilled water at 80℃ for 2 hr and filtered through filter paper. Then, 1 ml of the extract was mixed with 200 μl of CHCl_3_ and centrifuged at 8,791 *g* for 10 min. Afterward, 800 μl of each supernatant was filtered through a 0.2‐μm nylon filter prior to the HPLC analysis. The OPA regent (10 mg/ml) and borate buffer (0.4 M, pH= 10.4) used for derivatization were purchased from Agilent Technologies (Palo Alto, CA, USA). The online derivatization was developed using a SIL‐20A autosampler in accordance with the instructions provided in the Agilent Technical Note as follows: 5 µl of borate buffer, 0.5 µl of OPA buffer, and 1 µl of sample were drawn off successively, mixed nine times and then injected. The separation was completed using a Venusil AA column (4.6 × 250 mm, 5 μm, Chengdu, China). The mobile phases were solvents A (95 mM NaAc and 7% ACN, pH= 7.2) and solvent B (40% ACN, 40% MeOH, and 0.3% acetic acid). Elution conditions and flow rates were as follows: 0–5 min, solvent B was increased from 22% to 25% (linear gradient); 5–30 min, solvent B increased to from 25% to 100% (linear gradient); 30.0–32.9 min, solvent B was maintained at 100%; 33 min, solvent A increased from 0% to 22%, and then solvent A was maintained at 22% for 5 min. The flow rate was 1 mL/min. The temperature of the column oven was set at 40℃. The FLD was set at an excitation of 230 nm and an emission of 450 nm.

### 2.3 Widely‐targeted metabolomic experiments

Metabolites in the DOF and DDF were detected and examined using a widely targeted metabolomics approach, which was performed using UPLC‐MS/MS by Metware Biotechnology Co., Ltd. (www.metware.cn, Wuhan, China). The sample extraction, UPLC conditions, electron spray ionization (ESI)‐triple quadrupole‐linear ion trap mass spectrometry methods and metabolite data analysis were performed in accordance with their standard procedures and were previously fully described by Cao et al. (2019). A quality control (QC) analysis was conducted to confirm the reliability of the data. The QC sample was prepared by mixing sample into extracts, and it was inserted after every 10 samples to monitor changes in the repeated analyses.

### 2.4 Gas chromatography‐mass spectrometry (GC‐MS) analysis of medium‐ and long‐chain fatty acids

A GC‐MS approach was developed to measure the medium‐ and long‐chain fatty acids in DOF and DDF at Sanshu Bio‐technology Co., Ltd. (www.sanshubio.com, Nantong, Jiangsu, China). The reference fatty acids were obtained from Supelco (Bellefonte, PA, USA) and their standard curves are provided in Table S1. A 100‐mg flower sample was extracted with 4 ml of extraction buffer (1:1 chloroform:methanol, v/v) and 2 ml of 0.88% NaCl, and the mixture was vortexed for 30 s. Afterward, the mixture was centrifuged at 3500 rpm for 15 min, and then, the lower layer was transferred to a new Eppendorf tube. In total, 2 ml of CH_2_Cl_2_ was added to the tube and the sample was vortexed for 30 s and centrifuged at 748 *g* for 15 min. The lower layer was collected. The extraction with CH_2_Cl_2_ was repeated twice, and the lower layers of each extraction were combined and dried using nitrogen. The residue was first dissolved in 2 ml of MeOH containing of 1% H_2_SO_4_, vortexed for 30 s and then kept in an 80℃ water bath for 2 hr. After cooling to room temperature, 2 ml hexane and 1 ml desalted water were added, the mixture was vortexed for 30 s and then centrifuged at 2000 rpm for 5 min. The upper hexane phase was collected and 1 ml desalted water was added. The mixture was vortexed for 30 s and centrifuged at 244 *g* for 5 min. The upper phase was dried using nitrogen. After drying, 200 μl of isooctane was added to the residue, vortexed for 30 s, left standing for 5 min and then transferred to a GC glass injector insert for the GC‐MS analysis.

Fatty acids were analyzed on an Agilent 7890A GC‐MS equipped with an Agilent 7693 Autosampler and an Agilent 5975C inert XL EI/CI Mass Selective Detector (MSD) with Triple‐Axis Detector that was operated using Agilent MSD ChemStation software. Electron ionization at 70 eV was applied for fragmentation. An Agilent J&W CP‐Sil 88 FAME column (100 m × 0.25 mm, 0.20 µm) was used for separation. The chromatographic conditions were as follows: the temperature of the injection port was 250℃; helium was used as carrier gas at a flow rate of 1 ml/min; 2‐μl sample injection volume (splitless 10:1); and temperature program conditions of 100℃ (held for 5 min), then ramped at 4℃·min−1 to 240℃ (held for 15 min). Conditions used for the MS were as follows: transfer temperature of 260℃; scan range of 30–550 m/z; ionization potential of 70 eV; and electron multiplier voltage of 3000 V. The samples were analyzed in triplicate.

### 2.5 In vitro antioxidative activity assays

Flower powder (10 g) was extracted twice with 150 ml of 50% alcohol at room temperature for 48 hr and filtered. The extraction was concentrated using a rotary evaporator (IKA RV 10 Digital, IKA Laboratory Equipment, Staufen, Germany) at 40℃ under 0.08 MPa. The resulting concentrate was freeze‐dried to a powder using an FD5‐series Vacuum Freeze Dryer (GOLD‐SIM, Seattle, WA, USA). The antioxidative activities, including total antioxidant capacity and the scavenging of 1,1‐diphenyl‐2‐picrylhydrazyl (DPPH) and hydroxyl radicals, were evaluated using commercial kits (Grace Biotechnology Co., Ltd., Suzhou, China) in accordance with the manufacturer's instructions.

### 2.6 Statistical analysis

Each sample was extracted three times, and each extraction was detected twice. Statistical analyses were performed using Prism 8 for Windows (GraphPad Software, San Diego, CA, USA). Results are expressed as mean ± standard deviations (*n* = 6). All the data were analyzed using multiple *t*‐tests, and *p* < .05 was considered statistically significant. A principal component analysis (PCA) and a (orthogonal) partial least‐squares‐discriminant analysis (OPLS‐DA) were applied to comparison groups using SIMCA14.1 (Umetrics software, Malmö, Sweden). The variable importance in the projection (VIP) was used to rank the over‐all contribution of each variable in the OPLS‐DA model, and variables with VIP > 1.0, *p* < .05 and fold change (FC) ≥2 or FC ≤0.5 were considered as differential level metabolites for group discrimination.

## 3 RESULTS

### 3.1 Overview of Phytochemicals in DOF and DDF identified by the targeted metabonomics analysis

**FIGURE 1:**
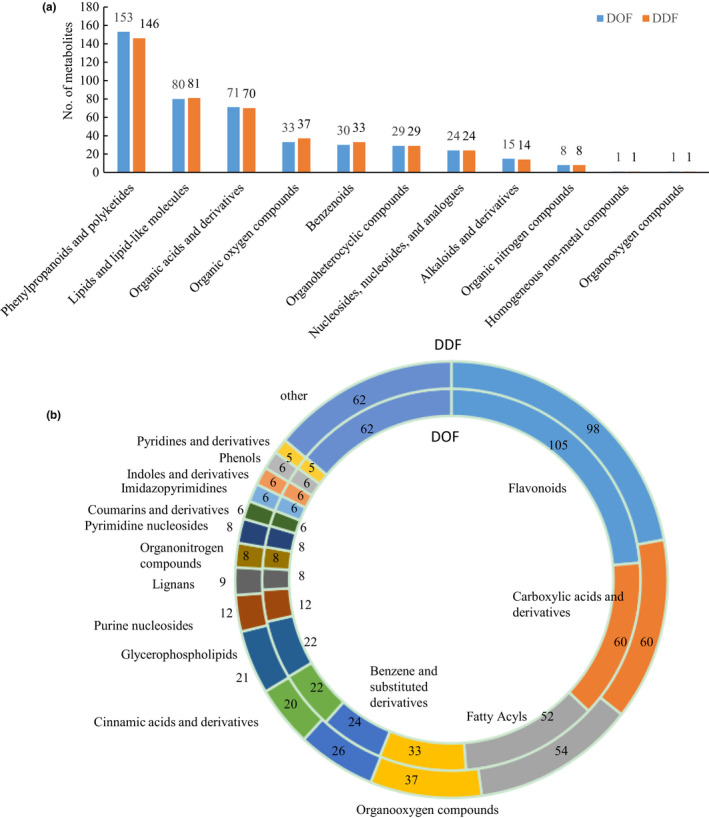
Superclasses (a) and classes (b) of metabolites identified in DOF and DDF

Using the widely targeted metabonomics method, 444 and 445 metabolites in DOF and DDF, respectively, were identified, for example, lysine, aspartic acid, and luteolin‐7‐O‐glucoside (Data S1: sheet 1). In total, these metabolites were grouped into 11 superclasses in accordance with The Human Metabolome Database classification system, and major were phenylpropanoids and polyketides (153 and 146 metabolites), lipids and lipid‐like molecules (80 and 81 metabolites), organic acids and derivatives (71 and 70 metabolites), organic oxygen compounds (33 and 37 metabolites), benzenoids (30 and 33 metabolites), organoheterocyclic compounds (29 metabolites), nucleosides, nucleotides and analogs (24 metabolites), alkaloids and derivatives (15 and 14 metabolites), and others in DOF and DDF, respectively (Figure 1a). They were further grouped into 54 classes, and major were flavonoids (105 and 98 metabolites), carboxylic acids and derivatives (60 metabolites), fatty acyls (52 and 54 metabolites), organooxygen compounds (33 and 37 metabolites), benzene and substituted derivatives (24 and 26 metabolites), cinnamic acids and derivatives (22 and 20 metabolites), glycerophospholipids (22 and 21 metabolites), purine nucleosides (12 metabolites), and lignans (8 and 9 metabolites) in DOF and DDF, respectively (Figure 1b; Data S1: sheet 2).

### 3.2 Amino acids in DOF and DDF

The free amino acid contents in DOF and DDF were 2.37% ± 0.07% and 1.64% ± 0.05%, respectively. Using the widely targeted metabonomics method, 59 metabolites grouped into amino acids, peptides and analogs were identified, including L‐aspartic acid, L‐leucine, L‐(−)‐tyrosine (Data S1: sheet 3). Using an HPLC analysis, 16 amino acids were detected in DOF and DDF (Table 1). In DOF, the contents of serine, at 1.51 ± 0.09 mg/g, and cysteine, at 1.09 ± 0.12, were highest; whereas in DDF, the contents of cysteine, at 1.38 ± 0.1 mg/g, and arginine, at 1.12 ± 0.06 mg/g, were the highest. These results were consistent with the reports of (Huang et al., 2017) and (Zhang et al., 2019), all showed flowers of *Dendrobium* are rich in amino acids, including seven essential amino acids.

**TABLE 1:** Contents of free amino acids in DOF and DDF (*n* = 6)

Amino acids	DOF (mg/g)	DDF (mg/g)
Alanine	0.08 ± 0.01	0.3 ± 0.03
Arginine	0.51 ± 0.09	1.12 ± 0.06
aspartic acid	0.19 ± 0.02	0.22 ± 0.01
Cystine	1.09 ± 0.12	1.38 ± 0.1
glutamic acid	0.07 ± 0.03	0.26 ± 0.01
Glycine	0.17 ± 0.02	0.34 ± 0.05
Histidine	0.42 ± 0.04	0.7 ± 0.03
Isoleucine	0.06 ± 0	0.17 ± 0.01
Leucine	0.18 ± 0.01	0.35 ± 0.02
Methionine	0.19 ± 0.02	0.46 ± 0.02
Valine	0.04 ± 0.02	0.05 ± 0
Phenylalanine	0.1 ± 0.01	0.28 ± 0.01
Proline	0.2 ± 0.4	0.1 ± 0
Serine	1.51 ± 0.09	0.53 ± 0.02
Threonine	0.04 ± 0	0.12 ± 0.01
Tyrosine	0.08 ± 0.01	0.22 ± 0.02

### 3.3 Lipids and lipid‐like molecules in DOF and DDF

**FIGURE 2:**
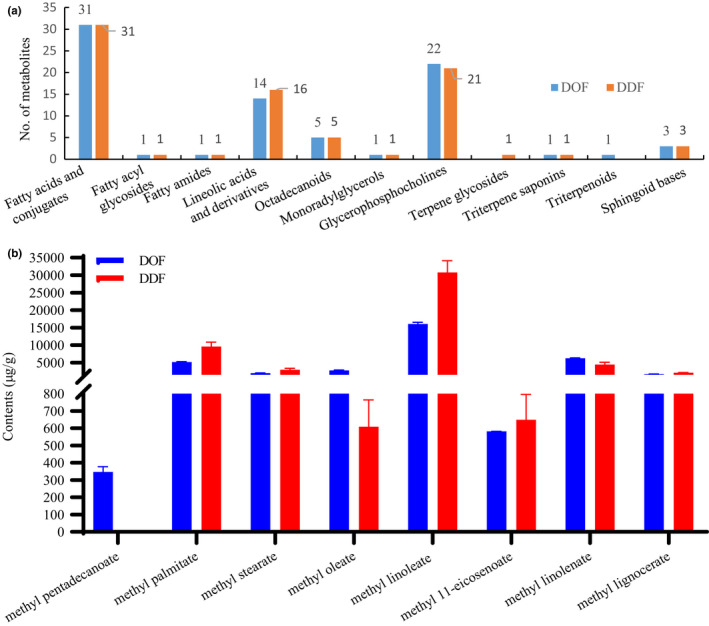
Classification of lipids and lipid‐like molecules in DOF and DDF (a), the contents of fatty acids detected in DOF and DDF (b)

In total, 80 and 81 identified metabolites were classified as lipids and lipid‐like molecules, and major were fatty acyls (52 and 54 metabolites), glycerophospholipids (22 and 21 metabolites) in DOF and DDF, respectively (Data S1: sheet 4). Among them, 31 compounds were further grouped as fatty acids or their conjugates, such as palmitoleic acid, stearic acid and eicosenoic acid. Additionally, 14 and 16 metabolites in DOF and DDF were grouped as linoleic acids and derivatives, such as α‐linolenic acid and *γ*‐linolenic acid, indicating that DOF and DDF contain diverse fatty acids (Figure 2a). To verify this, a GC–MS analysis for the determination of 36 medium‐ and long‐chain fatty acids in DOF and DDF was developed. Seven fatty acid methyl esters, methyl palmitate, methyl stearate, methyl oleate, methyl linoleate, methyl 11‐eicosenoate, methyl linolenate, and methyl lignocerate, were identified in both DOF and DDF, but methyl pentadecanoate was detected only in DOF. The methyl linoleate contents, at 16.09 ± 0.45 and 30.76 ± 3.40 mg/g, were the highest in both DOF and DDF. The methyl linolenate contents were 9.61 ± 1.26 and 4.44 ± 0.67 mg/g in the DOF and DDF flowers, respectively (Figure 2b). Linoleate protects against palmitate‐induced inflammation in cells (Maruyama et al., 2014), and dietary linoleate preserves cardiolipin and attenuates mitochondrial dysfunction in failing rat hearts (Mulligan et al., 2012). The *α*‐Linolenic acid is an essential fatty acid needed for human health that has cardiovascular‐protective, anticancer, neuroprotective, antiosteoporotic, anti‐inflammatory, and antioxidative effects (Kim et al., 2014). Therefore, metabolomics analyses showed that flowers of *Dendrobium* may be good sources of essential polyunsaturated fatty acids, such as linoleic and linolenic acids, which have various health benefits.

### 3.4 Flavonoids in DOF and DDF

**FIGURE 3:**
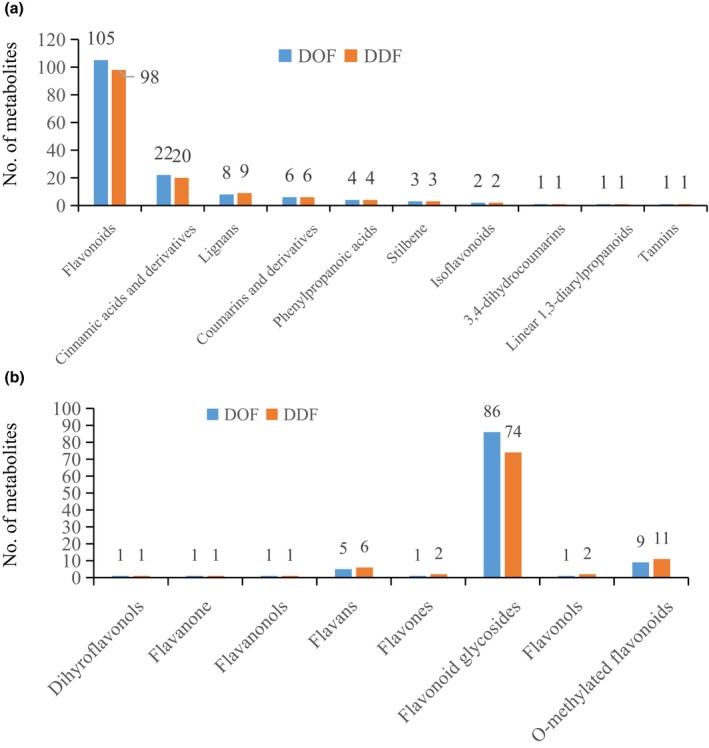
Classification of phenylpropanoids and polyketides (a) and flavonoids (b) identified in DOF and DDF

Spectrophotometric measurements identified the total polyphenols contents as 14.58 ± 0.39 and 9.89 ± 0.22 in DOF and DDF, respectively. In a previous work, we identified 21 phenolic compounds from DOF that were grouped into *O*‐glycosylflavones, *C*‐glycosylflavones, and phenylpropanoids (Zhang et al., 2019). In this metabonomics analysis, 153 and 146 metabolites grouped as phenylpropanoids and polyketides were identified. These metabolites were further characterized as flavonoids (105 and 98 metabolites), cinnamic acids and derivatives (22 and 20 metabolites), lignans (8 and 9), coumarins and derivatives (6), phenylpropanoic acids (4), and others (Figure 3a; Data S1: sheet 5). These flavonoids included quercetin and quercetin derivatives (18 metabolites), luteolin and luteolin derivatives (18 metabolites), apigenin glycosides (17 metabolites), kaempferol and kaempferol derivatives (13 metabolites) (Table 2), and major of them grouped as flavonoid glycosides (86 and 74 metabolites) and O‐methylated flavonoids (9 and 11 metabolites) in DOF and DDF, respectively (Figure 3b).

**TABLE 2:** Flavonoids in DOF and DDF

Contents of flavonoids measured by HPLC (mg/g)			Flavonoids identified in metabonomics analysis
	DOF	DDF	
Quercetin	1.13 ± 0.11	3.61 ± 0.76	Avicularin (Quercetin 3‐a‐L‐arabofuranoside), Bioquercetin, Di‐O‐methylquercetin, Hyperin (Quercetin 3‐galactoside), Quercetin 3,7‐bis‐O‐β‐D‐glucoside, Quercetin 3‐O‐glucoside, Quercetin 3‐O‐rhanosylgalactoside, Quercetin 3‐O‐β‐(2''‐O‐acetylβ‐D‐glucuronide), Quercetin 5‐O‐malonylhexosyl‐hexoside, Quercetin 7‐O‐malonylhexosyl‐hexoside, Quercetin O‐acetylhexoside, Quercetin‐3‐O‐glucoside‐7‐O‐rhamnoside, Quercetin‐3‐O‐α‐L‐arabinopyranoside, Quercetin‐O‐rutinoside‐hexose, Quercitrin, Rhamnetin (7‐O‐Methxyl Quercetin), Quercetin 3‐rutinoside, Quercetin 4'‐glucoside
Taxifolin	0.23 ± 0.08		Taxifolin
Rutin	0.44 ± 0.10	0.06 ± 0.09	Rutin
Luteolin	0.01 ± 0.03	0.06 ± 0.02	Diosmetin (Luteolin 4'‐methyl ether), Isoorientin (Luteolin‐6‐C‐glucoside), Lonicerin (Luteolin‐7‐O‐rhamnoside), Luteolin, Luteolin 3',7‐di‐O‐glucoside, Luteolin 8‐C‐hexosyl‐O‐hexoside, Luteolin C‐hexoside, Luteolin O‐hexosyl‐O‐pentoside, Luteolin‐6,8‐di‐C‐glucoside, Luteolin‐6‐C‐2‐glucuronylglucoside, Luteolin‐7,3'‐Di‐O‐β‐D‐Glucoside, Luteolin‐7‐O‐glucoside Luteolin‐7‐O‐rutinoside, Luteolin‐7‐O‐β‐D‐glucuronide, Luteolin‐7‐O‐β‐D‐rutinoside, Orientin (Luteolin 8‐glucoside), C‐Hexosyl‐luteolin C‐pentoside, C‐Hexosyl‐luteolin O‐sinapic acid
Kaempferol	0.24 ± 0.02	1.51 ± 0.31	6‐Hydroxykaempferol‐3,6‐O‐Diglucoside, 6‐Hydroxykaempferol‐3,7,6‐O‐triglycoside, 6‐Hydroxykaempferol‐3‐O‐rutin‐6‐O‐glucoside, 6‐Hydroxykaempferol‐7,6‐O‐Diglucoside, Astragalin (Kaempferol 3‐O‐glucoside), Dihydrokaempferol, Kaempferide 3‐O‐β‐D‐glucuronide, Kaempferol, Kaempferol 3‐O‐rutinoside, Kaempferol 3‐O‐β‐(2''‐O‐acetyl‐β‐D‐glucuronide), Kaempferol 7‐O‐glucosdie, Kaempferol 7‐O‐rhamnoside, Kaempferol‐3‐O‐glucoside‐7‐O‐rhamnoside
Myricetin	0.03 ± 0.03	0.65 ± 0.29	Taxifolin (Dihydromyricetin)
Others			(‐)‐Epiafzelechin, 5‐Hydroxyauranetin, 6‐C‐Hexosyl‐hesperetin O‐hexoside, 8‐C‐Hexosyl‐apigenin O‐feruloylhexoside, 8‐C‐Hexosyl‐apigenin O‐hexosyl‐O‐hexoside, 8‐C‐Hexosyl‐chrysoeriol O‐feruloylhexoside, 8‐C‐Hexosyl‐hesperetin O‐hexoside, Apigenin 5‐O‐glucoside, Apigenin 6,8‐C‐diglucoside, Apigenin 8‐C‐pentoside, Apigenin‐6‐C‐2‐glucuronylxyloside, Apigenin‐6‐C‐glucose‐8‐xylcose, Apigenin‐6‐C‐β‐D‐xyloside‐8‐C‐β‐Darabinoside, Apigenin‐7‐O‐(6'‐O‐acetyl)‐β‐D‐glucoside, Apigenin‐7‐O‐(6‐O‐Malonyl Glucoside), Butin, Catechin gallate, C‐Hexosyl‐apigenin O‐p‐coumaroylhexoside, C‐Hexosyl‐apigenin O‐pentoside, Chrysoeriol 5‐O‐hexoside, Chrysoeriol O‐malonylhexoside, Chysoeriol‐6,8‐di‐C‐glucoside, Cyanidin 3‐O‐galactoside, Cyanidin 3‐O‐glucoside, Cyanidin 3‐rutinoside, Di‐C,C‐hexosyl‐apigenin, Eriodictiol C‐hexosyl‐O‐hexoside, Eriodictyol, Eriodictyol C‐hexoside, Eupatilin 3‐glucoside, Genistein 8‐C‐glucoside, Gossypitrin, Herbacetin, Hesperetin 5‐O‐glucoside, Hispidulin, Isorhamnetin, Isorhamnetin 3‐O‐β‐(2''‐O‐acetyl‐β‐D‐glucuronide), Isorhamnetin acetyl hexoside, Isorhamnetin hexose‐malonate, Isorhamnetin O‐acetyl‐hexoside, Isorhamnetin‐3‐O‐rutinoside, Isoschaftoside, Isovitexin, Isovitexin 7‐O‐glucoside, Jaceosidin, Kaempferide 3‐O‐β‐D‐glucuronide, Ladanein, Naringenin, Nepetin, Peonidin 3‐O‐glucoside chloride, Pinobanksin, Quercitrin, Rhoifolin, Schaftoside, Tamarixetin, Tangeretin, Tricin 7‐O‐hexoside, Tricin 7‐O‐hexosyl‐O‐hexoside, Tricin O‐malonylhexoside, Tricin O‐saccharic acid, Tricin O‐sinapoylhexoside, Violanthin, Vitexin, Vitexin 2''‐O‐β‐L‐rhamnoside, Vitexin‐2‐O‐D‐glucopyranoside

To verify the flavonoid contents in DOF and DDF, six flavonoids, quercetin, taxifolin, rutin, luteolin, kaempferol, and myricetin, were further measured by HPLC (Table 2). Among them, quercetin had relatively high contents, at 1.13 ± 0.11 mg/g and 3.61 ± 0.76 in DOF and DDF, respectively. The kaempferol contents were 0.24 ± 0.02 mg/g and 1.51 ± 0.31 mg/g in DOF and DDF, respectively. The taxifolin, rutin, and myricetin levels ranged from 0 to 0.65 ± 0.29 mg/g. In a recently work, Huang identified 15 phenolic compounds in DOF, with the main phenolic compound being rutin at 0.14 ± 0.1 mg/g; however, the quercetin and kaempferol contents were lower than in our study (Huang et al., 2020). This may be because the extraction methods differed. We used methanol:hydrochloric acid (40:4, v/v) to extract and hydrolyze the quercetin and kaempferol glycosides, while Huang used 70% methanol only to extract free quercetin and kaempferol (Huang et al., 2020). Flowers of *Dendrobium* contain diverse flavonoids, especially quercetin, kaempferol and their derivatives. Quercetin derivatives have various biological activities, such as anticancer, antiviral and antioxidant (Magar & Sohng, 2020). Kaempferol possesses a wide range of therapeutic properties, such as antioxidant, anticancer and anti‐inflammatory (Imran et al., 2019). Therefore, flowers of *Dendrobium* appear to possess the biological activities of flavonoids and are worthy of further study.

### 3.5 Metabolite differences between DOF and DDF

**FIGURE 4:**
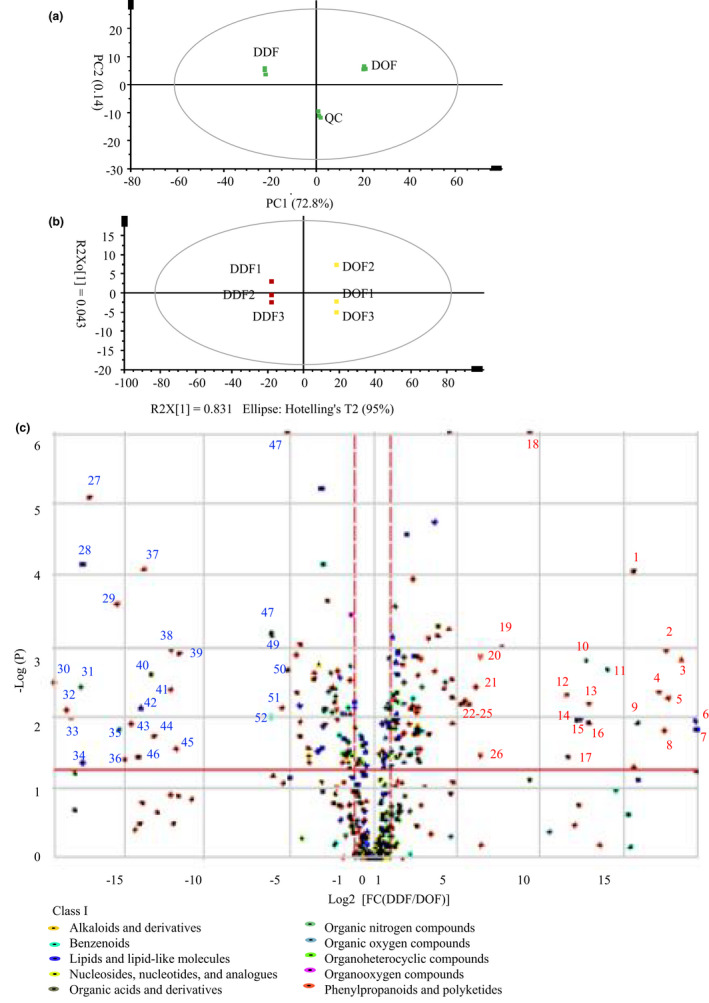
Principal component analysis (PCA) plots (a) and (Orthogonal) partial least‐squares‐discriminant analysis (OPLS‐DA) (b) analysis of metabolites. Volcano analysis of metabolites presents at different levels metabolites between DOF and DDF (c). NOTE: 1, Psoralenol; 2, 6‐Hydroxykaempferol‐3‐O‐rutin‐6‐O‐glucoside; 3,6‐Hydroxykaempferol‐3,6‐O‐Diglucoside. 4, Eriodictiol C‐hexosyl‐O‐hexoside; 5, Scopolin; 6, Quillaic acid; 7, Geniposidic acid; 8, Quercetin; 9, L‐(+)‐Tartaric acid; 10, Cimicifugamide; 11, 3‐Hydroxy‐4‐isopropylbenzylalcohol 3‐glucoside; 12, p‐Coumaraldehyde; 13, Herbacetin; 14, β‐D‐Furanofructosyl‐α‐D‐(3‐mustard acyl)glucoside; 15, MAG(18:3)isomer2; 16, 1‐Hydroxypineolin Diglucoside; 17, 7‐O‐Methxyl Quercetin; 18, Quercetin 7‐O‐malonylhexosyl‐hexoside; 19, 6‐Hydroxykaempferol‐3,7,6‐O‐triglycoside; 20, Luteolin‐7,3'‐Di‐O‐β‐D‐Glucoside; 21, Apigenin 7‐neohesperidoside; 22, 2,4,6,4'‐Tetrahydroxy‐stilbene‐2‐O‐D‐glucopyranoside; 23, Kaempferol 3‐O‐beta‐D‐galactoside; 24, Kaempferol 3‐O‐glucoside; 25, Luteolin‐7‐O‐β‐D‐glucuronide; 26, Kaempferol; 27, Peonidin 3‐O‐glucoside chloride; 28, 2‐Hydroxyoleanolic acid; 29, C‐Hexosyl‐luteolin C‐pentoside; 30, Violanthin; 31, Caffeoylcholine 5‐glucoside; 32, Chysoeriol‐6,8‐di‐C‐glucoside; 33, Luteolin‐6,8‐di‐C‐glucoside; 34, LysoPC(16:2); 35, Gallic acid; 36, C‐Hexosyl‐apigenin O‐p‐coumaroylhexoside; 37,C‐Hexosyl‐luteolin C‐pentoside; 38, Ethyl caffeate; 39,3,4‐Dimethoxycinnamic acid; 40,N‐Acetyl‐5‐hydroxytryptamine; 41, Eupatilin 3‐glucoside; 42, 6'‐O‐Sinapoyljasminoside B; 43,(+)‐Medioresinol‐ace Glu; 44, Tricin O‐sinapoylhexoside; 45, Tricin 7‐O‐hexosyl‐O‐hexoside; 46, Apigenin‐6‐C‐glucose‐8‐xylcose; 47, Trans‐arachidin‐caffeoyl‐rham; 48, Syringic acid; 49, Isorhamnetin acetyl hexoside; 50, Isorhamnetin hexose‐malonate; 51, Luteolin‐6‐C‐2‐glucuronylglucoside; 52, Syringic acid O‐glucoside

In the PCA score plot, QC samples clustered together, suggesting that this method had good stability and reproducibility (Figure 4a). Differential clustering of DOF and DDF metabolites in the PCA and OPLS‐DA indicated that they were significantly different (Figure 4a, b). Relative levels of 269 metabolites were significantly different between DDF and DOF (VIP > 1.0, *p* < .05 and FC < 0.5 or FC > 2), and the majority of these metabolites belonged to phenylpropanoids and polyketides, benzenoids and lipids and lipid‐like molecules (Figure 4c; Data S1: sheet 6).

The relative peak areas (PAs) of 144 metabolites in DDF were significantly greater than those in DOF (VIP > 1.0, *p* < .05 and FC > 2). Among them, 26 metabolite PAs were more than 33‐fold greater in DDF compared with DOF or they were only detected in DDF. They included psoralenol (1), 6‐hydroxykaempferol‐3‐O‐rutin‐6‐O‐glucoside (2), 6‐hydroxykaempferol‐3,6‐O‐diglucoside (3), eriodictiol C‐hexosyl‐O‐hexoside (4), scopolin (5), quillaic acid (6), quercetin (8) and kaempferol (26) (Figure 4c). The high levels of quercetin and kaempferol in DDF were in accordance with HPLC measurements. Thus, compared with in DOF, quercetin, kaempferol and their glycoside levels were relatively higher in DDF.

In total, the PAs of 125 metabolites in DDF were significantly smaller than those in DOF (VIP > 1.0, *p* < .05 and FC < 0.5), and among them, the PAs of 26 metabolites in DDF were more than 33‐fold smaller than in DOF, or they were only detected in DOF. They included peonidin 3‐O‐glucoside chloride (27), 2‐hydroxyoleanolic acid (28), C‐Hexosyl‐luteolin C‐pentoside (29), caffeoylcholine 5‐glucoside (31), chysoeriol‐6,8‐d‐C‐glucoside (32), luteolin‐6,8‐di‐C‐glucoside (33), and lysoPC(16:2) (34) (Figure 4c). Interestingly, (Zhang et al., 2019) identified 11 C‐glycosylflavones in DOF, and (Zhou et al., 2012) found that the flavone C‐glycoside contents in leaves and flowers were greater than in stems of *D. officinale*. We found that the PAs of C‐glycosides, including C‐hexosyl‐luteolin C‐pentoside (29), chysoeriol‐6,8‐di‐C‐glucoside (32), luteolin‐6,8‐di‐C‐glucoside (33),C‐hexosyl‐luteolin C‐pentoside (37), apigenin‐6‐C‐glucose‐8‐xylcose (46) and luteolin‐6‐C‐2‐glucuronylglucoside (51), in DOF were greater than in DDF. Thus, C‐glycosides appear to be characteristic compounds of DOF.

### 3.6 Antioxidative activities of DOF and DDF extracts

**FIGURE 5:**
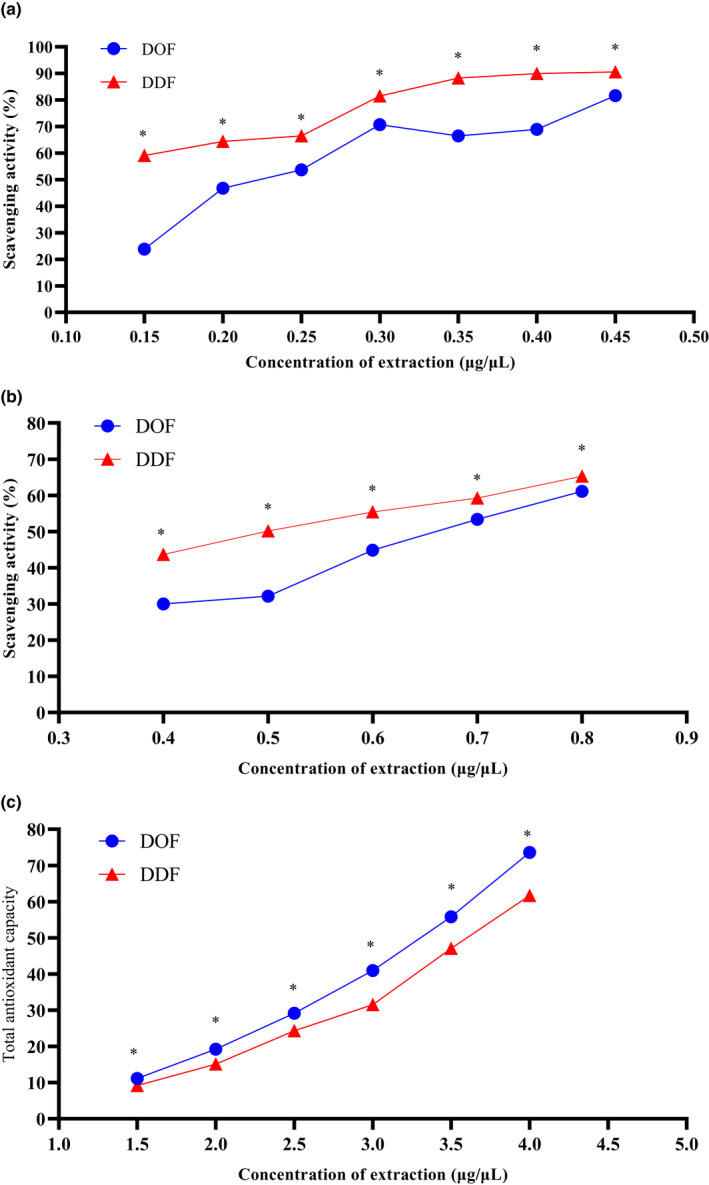
The scavenging capabilities of 1,1‐diphenyl‐2‐picrylhydrazyl (DPPH·) (a) and hydroxyl radicals (OH·) (b) of extracts of DOF and DDF. The total antioxidant capacities of extracts of DOF and DDF (c). *Indicates a significant difference in activity between extracts of DOF and DDF at the same concentration (*n* = 6)

Both HPLC and metabonomics analyses identified flavonoids, including quercetin, kaempferol and their glycosides, in DOF and DDF. These flavonoids have antioxidative activities; therefore, we measured the antioxidative activities of DOF and DDF extracts using in‐vitro assays. Both DOF and DDF extracts scavenged DPPH (Figure 5a) and hydroxyl radicals (Figure 5b), and they had dose‐dependent total antioxidant capacities (Figure 5c). The DDF extract exhibited significantly greater DPPH and hydroxyl radical scavenging capabilities than the DOF extract (*p* < .05). Additionally, the total antioxidant capacity of the DOF flower extract was greater than that of the DDF extract (*p* < .05). The antioxidative activities of DOF, such as DPPH (Zhang et al., 2019), hydroxyl (Liao et al., 2019) and 2,2′‐azino‐bis (3‐ethylbenzothiazoline‐6‐sulfonate) (He et al., 2016; Liang et al., 2018; Liao et al., 2019) radical scavenging capacities have been reported previously. DOF also appear to have antihypertensive effects (Li et al., 2019; Li et al., 2020), alleviate brain aging and improve the spatial learning abilities of senescent rats (He et al., 2017). However, this is the first report on the phytochemicals and antioxidative activities of DDF. Because both DOF and DDF are rich in phytochemicals, their biological activities are worth further study to explore their applications in functional foods and pharmaceuticals.

## 4 CONCLUSION

In conclusion, using metabonomics, HPLC and GC‐MS, we revealed that DOF and DDF contained amino acids, and diverse flavonoids, especially quercetin, kaempferol, and their derivatives, which are rich in methyl linoleate and methyl linolenate. Extracts of both DOF and DDF presented high antioxidative activities. Thus, we further characterized the phytochemicals in DOF, and presented the first report on the phytochemicals and antioxidative activities of DDF. This research increased our knowledge of these edible flowers and may increase their popularity among consumers and lead to applications in functional foods and pharmaceuticals.

## CONFLICTS OF INTEREST

The authors declared no potential conflicts of interest with respect to the research, authorship, and publication of this article.

## AUTHOR CONTRIBUTIONS

Ming Zhao. and Xingfen Zhang involved in conceptualization, methodology, data curation, formal analysis, investigation, and writing the original draft. Qianting Liu and Congpin Li involved in conceptualization, funding acquisition, methodology, supervision. Jiakun Fan and Hui Luo involved in conceptualization and resources. Jurun Zhao and Qinyan Tang involved in investigation.

## Supporting information

Table S1

Data S1

## Data Availability

The data that support the findings of this study are available on request from the corresponding author. The data are not publicly available due to privacy or ethical restrictions.
