# Supplementary material for: Phytochemical profiles of edible flowers of medicinal plants of Dendrobium officinale and Dendrobium devonianum
Source: Food Sci Nutr. 2021 Oct 4;9(12):6575–86. doi: 10.1002/fsn3.2602 (PMC8645735; doi:10.1002/fsn3.2602)
Supplement: Supplementary file 1 — Table S1 [file FSN3-9-6575-s002.docx]

**Table S1** refence fatty acids and standard curves for GC-MS analysis

| Number | Fatty acids | CAS ID | Standard curves | **R^2^** |
| --- | --- | --- | --- | --- |
| 1 | Methyl Hexanoate | 106-70-7 | y = 14714.536916 * x + 286793.308259 | 0.998 |
| 2 | Methyl Octanoate | 111-11-5 | y = 20577.281435 * x + 193685.547683 | 0.999 |
| 3 | Methyl Decanoate | 110-42-9 | y = 22702.861875 * x + 209397.877794 | 0.999 |
| 4 | Methyl Undecanoate | 1731-86-8 | y = 23078.439359 * x + 208511.421097 | 0.999 |
| 5 | Methyl Laurate | 111-82-0 | y = 23383.146314 * x + 210108.326353 | 0.998 |
| 6 | Methyl Tridecanoate | 1731-88-0 | y = 23340.657237 * x + 199510.726967 | 0.998 |
| 7 | Methyl Myristate | 124-10-7 | y = 23337.518587 * x + 207819.932538 | 0.998 |
| 8 | Methyl Myristoleate | 56219-06-8 | y = 8740.105869 * x + 56009.802010 | 0.999 |
| 9 | Methyl Pentadecanoate | 7132-64-1 | y = 22247.516870 * x + 223474.323115 | 0.998 |
| 10 | Methyl Pentadecenoate | 90176-52-6 | y = 9377.221286 * x + 61607.722092 | 0.999 |
| 11 | Methyl Palmitate | 112-39-0 | y = 19863.031235 * x + 529405.318390 | 0.998 |
| 12 | Methyl Palmitoleate | 1120-25-8 | y = 7410.548772 * x + 47682.774448 | 0.999 |
| 13 | Methyl Heptadecanoate | 1731-92-6 | y = 21169.963858 * x + 197513.387939 | 0.998 |
| 14 | Methyl Heptadecenoate | 31424-16-5 | y = 7956.815803 * x + 56314.181566 | 0.999 |
| 15 | Methyl Stearate | 112-61-8 | y = 20639.242626 * x + 182425.098644 | 0.999 |
| 16 | Methyl Elaidate | 2462-84-2 | y = 7023.874129 * x + 46684.692196 | 0.999 |
| 17 | Methyl Oleate | 112-62-9 | y = 6968.881849 * x + 56156.496101 | 0.999 |
| 18 | Methyl Linoelaidate | 2566-97-4 | y = 8708.375561 * x + 37854.736150 | 0.999 |
| 19 | Methyl Linoleate | 112-63-0 | y = 8649.440184 * x + 51822.866558 | 0.999 |
| 20 | Methyl Arachidate | 1120-28-1 | y = 19201.489088 * x + 134692.947946 | 0.999 |
| 21 | Methyl Gamma Linolenate | 16326-32-2 | y = 8209.851702 * x + 38553.655482 | 0.999 |
| 22 | Methyl 11-Eicosenoate | 3946-8-5 | y = 7295.512993 * x + 46628.700359 | 0.999 |
| 23 | Methyl Linolenate | 301-00-8 | y = 10213.483230 * x + 42671.193932 | 0.999 |
| 24 | Methyl Heneicosanoate | 6064-90-0 | y = 17893.089434 * x + 134248.443908 | 0.998 |
| 25 | Methyl 11,14-Eicosadienoate | 2463-2-7 | y = 8445.278815 * x + 39907.498260 | 0.998 |
| 26 | Methyl Behenate | 929-77-1 | y = 17604.853236 * x + 51726.843368 | 0.998 |
| 27 | cis-8,11,14-Eicosatrienoic acid methyl ester | 21061-10-9 | y = 7835.334197 * x + 22710.010850 | 0.998 |
| 28 | Methyl Erucate | 1120-34-9 | y = 7108.552793 * x + 19045.177844 | 0.999 |
| 29 | Methyl 11,14,17-Eicosatrienoate | 55682-88-7 | y = 9343.759367 * x + 17799.344545 | 0.999 |
| 30 | Methyl Tricosanoate | 2433-97-8 | y = 12794.467384 * x + 27094.478709 | 0.999 |
| 31 | Methyl Arachidonate | 2566-89-4 | y = 7670.562236 * x + 294.954554 | 0.999 |
| 32 | Methyl Docosadienoate | 61012-47-3 | y = 7965.295868 * x - 18366.651361 | 0.998 |
| 33 | Methyl Lignocerate | 56219-06-8 | y = 15951.115977 * x - 378969.386562 | 0.999 |
| 34 | Methyl Eicosapentaenoate | 2734-47-6 | y = 8841.984141 * x + 1862.611979 | 0.999 |
| 36 | Methyl Nervonate | 2733-88-2 | y = 6963.758763 * x - 89165.518737 | 0.998 |
| 36 | Methyl Docosahexaenoate | 28061-46-3 | y = 7740.931957 * x - 94012.012820 | 0.998 |
